# Preeclampsia and Venous Thromboembolism: Pathophysiology and Potential Therapy

**DOI:** 10.3389/fcvm.2022.856923

**Published:** 2022-03-07

**Authors:** Tiphaine Raia-Barjat, Osasere Edebiri, Fionnuala Ni Ainle

**Affiliations:** ^1^Department of Gynecology and Obstetrics, Centre Hospitalier Universitaire de Saint-Étienne, Saint-Étienne, France; ^2^INSERM U1059, SAINBIOSE, Université Jean Monnet, Saint-Étienne, France; ^3^Department of Haematology, Mater Misericordiae University Hospital, Rotunda Hospital, Dublin, Ireland; ^4^UCD School of Medicine, University College Dublin, Dublin, Ireland

**Keywords:** preeclampsia, PET, pregnancy, thrombosis, risk

## Abstract

Preeclampsia (PET) is a multisystem inflammatory disorder that represents a leading cause of feto-maternal morbidity and mortality, complicating 2–5% of all pregnancies. PET incurs an increased risk of venous thromboembolism, which is one of the leading causes of death in pregnancy and in the postpartum period. This prothrombotic phenotype is attributable to the maternal phase of PET, which is characterized by a systemic inflammatory response and coagulation activation. Research continues to be undertaken in terms of preventative measures, however, currently revolves around pharmacological low dose aspirin initiated in the first trimester of pregnancy for those with risk factors. Treatment involves antenatal corticosteroids for fetal lung development in preterm birth, parenteral magnesium sulfate for fetal neuroprotection and maternal seizure prophylaxis, and timely birth of the fetus and placenta being the only definitive treatment of PET. Patients with a venous thromboembolism (VTE) risk deemed to be >1–3% are treated with pharmacological thromboprophylaxis in the form of low molecular weight heparin. Completing each woman’s VTE risk assessment is crucial, particularly in the setting of PET, as there is also a proven associated competing hemorrhagic risk.

## Introduction

Preeclampsia (PET) complicates 2–5% of all pregnancies and represents a leading cause of feto-maternal morbidity and mortality worldwide (1–3). PET is a multi-system inflammatory disorder and is estimated to account for 15% of maternal mortality worldwide (3–5). The classical clinical presentation of PET consists of the new onset of hypertension and proteinuria after 20 weeks gestation or other maternal organ dysfunction (6–8). Complications of PET include intra-uterine growth restriction (IUGR), fetal death (1–2% of cases), preterm birth, hepatic and renal dysfunction, thrombosis, coagulopathy, eclampsia (a severe manifestation of PET characterized by severe hypertension and generalized seizures) and maternal death (up to 70,000 deaths annually worldwide) (8–10).

Risk factors for PET include history of PET, chronic hypertension, pregestational diabetes mellitus, multiple pregnancy, obesity, and antiphospholipid syndrome (11, 12). Women with a history of VTE were also at increased risk of placenta-mediated complications (13). PET pathophysiology is considered to occur in two stages: abnormal placentation in the first trimester followed by maternal endothelial dysfunction in the second trimester (14). Crucially, hypertensive disorders in pregnancy are associated with a higher risk of arterial cardiovascular diseases (myocardial infarction and ischemic stroke) in later life (15–17). Moreover, PET is characterized by alterations in pro and anticoagulant pathways (18), beyond the physiological hypercoagulable state that occurs in pregnancy (19, 20). This hypercoagulable state may increase venous thromboembolism (VTE) risk (1), a major contributor to maternal morbidity and mortality (21–24). VTE is therefore not only a risk factor but also a consequence of PET (13).

## Pathophysiology of Preeclampsia

**FIGURE 1:**
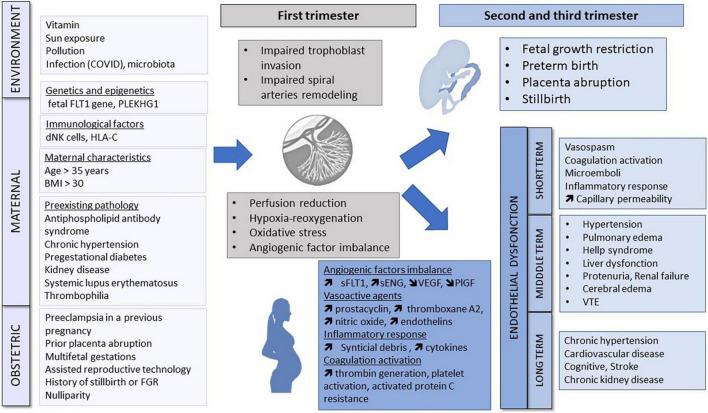
Pathogenesis of pre-eclampsia. Environment, genetic and epigenetic, immunological, maternal and obstetric factors may cause abnormal placentation in the first trimester. Placental ischemia leads to the release of antiangiogenic factors, vasoactive agents, inflammatory response and coagulation activation responsible for endothelial dysfunction and maternal organs dysfunction. FLT1, Fms-like tyrosine kinase; dNK, decidual natural killer; BMI, body mass index; FGR, fetal growth restriction; sFLT1, soluble fms-like tyrosine kinase 1; sENG, soluble endoglin; VEGF, vascular endothelial growth factor; PlGF, placental growth factor; VTE, Venous thromboembolism.

Preeclampsia pathophysiology is considered to occur in two stages in the first trimester and 2nd/3rd trimester (Figure 1).

Under normal physiological circumstances, the uteroplacental arteries are invaded by endovascular trophoblasts. The caliber of the spiral arteries widens, which facilitates a progressive increase of uteroplacental blood flow; and the tunic of the artery becomes toneless without maternal vasomotor control (25, 26). In PET, placental histology is characterized by impaired trophoblast invasion and failure of vascular remodeling (27). Although hypotheses have been proposed, underlying mechanisms remain poorly characterized. *Reduced oxygen tension and persistent hypoxia* appear to play an important role (28). With impaired spiral artery remodeling, trophoblast cells are exposed to a chronic intermittent hypoxia and reoxygenation phenomenon (29), leading to oxidative stress. *Oxidative stress* is associated with antioxidant depletion, oxidative damage and an inflammatory response (30, 31). *Immune mechanisms* at the maternal–placental interface may be multifactorial, involving a deficiency of natural killer cells at the beginning of placentation (32), and abnormal allorecognition of paternal HLA-C by the maternal killer Ig-like receptors (33). *Imbalances of angiogenic factors* have also been postulated to play a role, in particular vascular endothelial growth factor (VEGF) which plays a role in vascular remodeling (34). Overall PET heritability is estimated at 55%, with 30–35% maternal and 20% fetal *genetic* contributions to risk (35, 36). Emerging mechanisms hypothesized also to play a pathophysiological role include *epigenetic factors*, including dysregulation at the Fms-like tyrosine kinase 1 locus in the fetal genome (37, 38) or a maternal genome-wide susceptibility locus at rs9478812, which is an intronic region of protein PLEKHGI implicated in blood pressure regulation (39). These myriad pathogenetic processes may also be affected by maternal pre-existing characteristics, environmental and physiological factors (40, 41). It is plausible that a combination of mechanisms interact to initiate early changes that result in the clinical spectrum of PET.

Circulating factors that enter the maternal circulation as a consequence of abnormal placentation interact with endothelial cells, stimulating structural and functional changes that include altered vascular reactivity to vasomodulator substances, activation of the coagulation cascade and an increase in capillary permeability (14, 42, 43). Hypertension develops as a consequence of the maternal response to antiangiogenic factors, vasospasm and agonistic autoantibodies that bind to the angiotensin II type 1 receptor (AT1-AAs) (44). In the maternal preeclamptic circulation, excess levels of antiangiogenic factors including soluble Fms-like tyrosine kinase 1 (sFLT1) and soluble endoglin (sENG), coupled with a decrease in physiological levels of proangiogenic proteins including VEGF and placental growth factor (PlGF) result in an overall antiangiogenic state. These markers are used clinically during PET screening in the first trimester, and later as diagnostic or prognostic biomarkers (42, 45–51). The International Federation of Gynecology and Obstetrics (FIGO) recommend the use of this biomarkers in a first- trimester “screen and prevent” strategy for PET (52). Preeclamptic women exhibit a vasoconstrictive state secondary to the release of vasoactive agents such as prostacyclin, thromboxane A2, nitric oxide, and endothelins. Moreover, PET is also a proinflammatory state secondary to (1) systemic release of apoptotic and necrotic trophoblastic placental debris (53), (2) dysregulation in the balance of IL-10 and proinflammatory cytokines including IL-12 and IL-18 (54), and to (3) elevated complement level (55).

Collectively, these processes lead to systemic vascular and maternal organ dysfunction with long-term cardiovascular (56), cognitive (57) and renal (58) effects.

## Thromboembolic Risk and Preeclampsia

Venous thromboembolism (VTE) remains a leading cause of death in pregnancy and in the postpartum period (59). During 2014–2016, VTE was reported to be the top cause of direct maternal death in the United Kingdom and Ireland, occurring in 1.39 (95% CI 0.95–1.96) per 100,000 pregnancies (60). Women diagnosed with PET are reported to have a variable VTE risk, depending on their pregnancy stage (the highest-risk phase being the postpartum period) and PET severity (likely due to balanced alterations in pro and anticoagulant pathways). However, under some circumstances, women may have an up to five-fold increased risk of VTE compared to the normal pregnancy-associated VTE risk reported in the population (10).

Under normal physiological circumstances, pregnancy is characterized by the development of a hypercoagulable state, characterized by an increase in procoagulant factor activity and a down-regulation of endogenous anticoagulant and fibrinolytic pathways. It is postulated that this hypercoagulable state develops to limit the risk of major bleeding associated with labor and birth (61, 62). Although this pregnancy-associated hypercoagulability may reduce the risk of major peripartum bleeding, the shift toward a procoagulant phenotype also confers an increased risk of VTE.

This baseline pregnancy-associated elevated thromboembolic risk is increased in the presence of additional VTE risk factors. These risk factors may pre-date pregnancy, arise during pregnancy or occur peripartum, highlighting the crucial importance of performing a VTE risk assessment at several times during pregnancy and at labor and birth. A Norwegian register-based case-control study including 600,000 pregnancies reported a four-fold increased risk of VTE in patients with PET in the postpartum period, however, no association was identified between VTE and PET in antepartum period (63). These results are supported by several studies that reported similar results, assigning greatest VTE risk to the postpartum period (64); mechanisms underlying this observation are not fully characterized (10). Nevertheless PET is still considered as a risk when deciding if a woman needs antenatal thromboprophylaxis in the Royal College of Obstetricians and Gynaecologists (RCOG) guideline (65). An additive effect on the overall postpartum VTE risk was associated with PET complicated by intrauterine growth restriction (IUGR), incurring a seven-fold increased risk (66). In addition, the extent of hemostatic derangement and hypercoagulability appears to be further exacerbated by disease severity and stage; with early-onset PET (EOP) (onset before 34-weeks gestation) having an observed risk of a more severe phenotype (67).

Mechanisms which may underly this prothrombotic phenotype can be attributed to the maternal phase of PET which is characterized by a systemic inflammatory response accompanied by coagulation activation (10). The increased risk of VTE is thought to be multifactorial, involving endothelial dysfunction, coagulation and platelet activation among others (10).

Under normal physiological conditions, the endothelium includes an intact, negatively charged, and non-adhesive glycosaminoglycan layer which acts to inhibit thrombin generation and the adhesion of platelets and leucocytes (68). This endothelial layer expresses a number of anticoagulant proteins such as thrombomodulin (TM), the endothelial protein C receptor (EPCR), and tissue plasminogen activator (tPA) (69). Endothelial dysfunction and damage is extensively reported in PET, and may contribute to impaired activated protein C anticoagulant activity at the endothelial surface and increased exposure of sub-endothelial tissue factor, which is the primary activator of blood coagulation. This, coupled with increased expression of adhesion molecules such as ICAM-1, is postulated to promote the adhesion of inflammatory cells and increased release of endothelial extracellular vesicles (EVs). EVs have also been shown to have a pro-inflammatory and prothrombotic effect activating several pathological signaling pathways on leucocytes, neutrophils, and platelets. Placental-derived factors in PET appear to be key pathological mediators in the process of endothelial damage (67).

Aside from endothelial dysfunction, relative to normal pregnancy, PET is characterized by alterations in circulating platelet-derived microparticle (MP) and extracellular vesicle (EV) profiles, which may contribute to the PET-associated VTE risk, although a proven mechanistic association has not yet been defined (67).

## Preventive and Curative Treatment for Preeclampsia

Preeclampsia prevention and treatment continues to be investigated in ongoing studies. Simpler approaches have explored hygienic and dietetic strategies. Measures including bed rest (70), sodium restriction (71), folic acid (72), antioxidant (combined vitamin C and E therapy) (73), fish oil (74), and garlic (75) have failed to demonstrate a clinical benefit. Studies have suggested that exercise (76), and vitamin D (77) supplementation may reduce the PET, however, these studies are hampered by severe methodological limitations and a beneficial effect for these measures has not been proven. A Cochrane review suggests that in areas with a low calcium intake, high-dose calcium supplementation halves the risk of PET (78). Although there are some limitations to the evidence, the World Health Organization endorses the use of supplemental elemental calcium for pregnant women to reduce the PET risk.

Currently, PET prevention centers around low dose aspirin. In 2019, a Cochrane meta-analysis of 77 trials (40,249 women) determined that the risk of pre-eclampsia was 18% lower with low dose aspirin (95% CI, 12–23%) (79). In the ASPRE trial, aspirin 150 mg daily was administered to pregnant women at high-risk of pre-eclampsia as defined by a screening algorithm consisting of clinical, imaging and blood parameters (80). This trial reported a 62% reduction of the risk of pre-term PET and a 28% reduction in the combination of pre-term and term PET. In the recently published ASPIRIN randomized control trial (RCT), low-dose aspirin was commenced between 6 and 13 + 6 weeks of pregnancy and continued until 36 + 6 weeks. A significant reduction in the incidence of preterm birth before 37 weeks in nulliparous women was observed (RR 0.89, 95% CI, 0.81–0.98), along with reduced birth before 34 weeks in women with hypertensive disorders of pregnancy (RR 0.38, 95% CI, 0.17–0.85). Moreover, perinatal mortality (RR 0.86), fetal loss (RR 0.86), and early preterm birth before 34 weeks (RR 0.75) was also reduced (81).

Optimal timing of initiation and dose remain uncertain (82). Most evidence supports earlier initiation of aspirin prior to 20 weeks’ gestation and ideally prior to 16 weeks at (83, 84). Some authors suggest that aspirin administered at bedtime is more efficacious than awakening administration but this concept has not been included in all the recommendations (84–86). The combination of aspirin with low molecular weight heparin (LMWH) is not more efficient than aspirin alone in pregnant women with previous severe preeclampsia diagnosed before 34 weeks of gestation to prevent PET recurrence (87) without maternal or neonatal side effects.

Determining which women should be started on aspirin prophylactically is very challenging. Current evidence shows that no single test predicts pre-eclampsia with sufficient accuracy to be clinically useful (88), and thus signifies the need for improved risk stratification tools.

Preeclampsia without severe features is managed expectantly until 37 weeks, in the presence of severe features in those <34 weeks it may be managed expectantly with birth indicated at any time with deterioration of fetal and maternal status. The pharmacological management of mild to moderate hypertension (systolic <160 and diastolic <110) is not currently recommended by the ACOG, as it does not appear to attenuate disease progression and may increase the risk of fetal growth restriction. As this mild to moderate hypertension may be associated with a 4% risk of stroke, its treatment is still subject to debate (89). Treatment currently revolves around antenatal corticosteroids for fetal lung development in patients <34 weeks, and parenteral magnesium sulfate for fetal neuroprotection and maternal seizure prophylaxis; with timely birth of the fetus and placenta remaining the only definitive treatment of PET (12). The efficacy of magnesium sulfate to prevent seizures in women with preeclampsia with severe features and eclampsia is proven but is more debated in cases of moderate preeclampsia (83, 90, 91).

## Prevention of Thromboembolic Risk in Preeclampsia

Despite the fact that pre-eclampsia complicates a significant number of pregnancies and is the leading cause of morbidity and mortality in pregnancy, therapeutic strategies remain poorly characterized (10). The elevated baseline pregnancy-associated VTE risk is further increased by additional maternal, pregnancy and birth characteristics (such as PET) (9, 21, 92–98), highlighting the importance of VTE risk assessment to detect risk factors in early pregnancy, at birth and if risk factors change (65). VTE risk assessment protocols are based on the cumulative presence of multiple risk factors, of which preeclampsia is one component. Guidelines suggest consideration of thromboprophylaxis, particularly in the postnatal period and in the context of additional risk factors such as early onset PET and intrauterine growth retardation, when the overall VTE risk is >1–3% (99). Currently, pharmacological thromboprophylaxis, when it is indicated, is typically achieved through administration of low molecular weight heparin (67). Patient selection is determined based on VTE risk assessment, that should be conducted antepartum and postpartum. However, data supporting the optimal risk threshold at which thromboprophylaxis should be instituted, along with the optimal duration of anticoagulation are lacking, despite how commonly VTE risk factors in the postpartum period arise. As a broad principle, the benefit of pharmacological VTE prophylaxis should outweigh the risk of bleeding and other fetal complications (100). Completing each woman’s VTE risk assessment is crucial, particularly in the setting of pre-eclampsia as there is also a proven associated competing hemorrhagic risk. A nationwide cohort study in the Netherlands, reported that 7.4% of woman with pre-eclampsia developed postpartum hemorrhage, compared to 4.2% in those without pre-eclampsia (101). Therefore, determining which patients are more likely to be affected by bleeding complications is of great importance, and not fully elucidated.

The authors of a 2014 Cochrane review concluded that “there is insufficient evidence on which to base recommendations for thromboprophylaxis during pregnancy (and that) large scale, high-quality randomized trials of currently used interventions are warranted” (102). However, the experience of the PROSPER investigators has demonstrated that conducting RCTs for women with (in this case, postpartum) VTE risk factors can prove extremely challenging (103, 104).

Consequently, to date, guideline recommendations are mainly based on expert opinion rather than high-quality evidence (65, 99, 105–107). This can be extremely challenging for care providers, particularly given the competing risks and challenges of pharmacological thromboprophylaxis, which are relatively common and include bleeding, bruising, skin reactions, pain, and in many jurisdictions, high out-of-pocket costs. Data published to date suggest that women who have a strong thrombophilia or a history of previous VTE are likely to benefit from postpartum thromboprophylaxis. However, guideline recommendations regarding thromboprophylaxis strategies for women with more commonly occurring risk factors such as PET vary widely, with much controversy, in light of uncertainty regarding the optimal strategy. This knowledge gap is currently being addressed by the pilot PARTUM randomized controlled trial (Postpartum Aspirin to Reduce Thromboembolism Undue Morbidity; NCT04153760), a pilot trial that will evaluate the feasibility of conducting a larger multinational trial, in which postpartum women with VTE risk factors will be randomized to low-dose aspirin daily or placebo for 6 weeks.

## Discussion

Both PET and VTE remain a leading cause of maternal morbidity and mortality, complicating a significant number of pregnancies (2, 54). Underlying pathophysiological mechanisms modulate the baseline hypercoagulable state of pregnancy, influencing both pro and anticoagulant pathways such that some women exhibit and overall increased procoagulant state relative to normal pregnancy, particularly in the post-partum period (19, 20). Despite this fact, therapeutic strategies remain poorly characterized (8). Urgent research priorities include personalized risk prediction for PET development and PET-associated VTE risk along with continued refinement of PET prevention strategies. Addressing these knowledge gaps has the potential to result in reduced morbidity and mortality for both mothers affected by PET and their infants.

## Author Contributions

TR-B and OE wrote the sections of the manuscript. FN revised the manuscript. All authors contributed to manuscript revision, read, and approved the submitted version.

## Conflict of Interest

The authors declare that the research was conducted in the absence of any commercial or financial relationships that could be construed as a potential conflict of interest. The handling editor declared a shared affiliation with one of the authors, TR-B, at the time of review.

## Publisher’s Note

All claims expressed in this article are solely those of the authors and do not necessarily represent those of their affiliated organizations, or those of the publisher, the editors and the reviewers. Any product that may be evaluated in this article, or claim that may be made by its manufacturer, is not guaranteed or endorsed by the publisher.
